# Necessity of Distinguishing Verrucous Carcinoma From Verrucous Skin Lesion Overlaying Residual Skin Staples in an Area of Sensory Loss: A Case Report

**Published:** 2015-06-17

**Authors:** Shunsuke Sakakibara, Takahiro Tokiyoshi, Kazunobu Hashikawa, Hiroto Terashi

**Affiliations:** ^a^Department of Plastic Surgery, Kobe University Graduate School of Medicine, Kobe, Japan; ^b^Department of Plastic Surgery, National Hospital Organization Shikoku Cancer Center, Matsuyama, Japan

**Keywords:** diabetic foot, diabetic neuropathy, verrucous carcinoma, verrucous skin lesion, VSLDN

## Abstract

**Objective:** Verrucous skin lesions on the feet in diabetic neuropathy is a condition usually induced by chronic mechanical stimulation of the feet of patients with diabetic neuropathy and usually occurs at weight-bearing sites. We here present a rare case involving a man with verrucous skin lesions on the feet in diabetic neuropathy at an unusual, non–weight-bearing site. **Methods:** A 58-year-old man with diabetic neuropathy presented with a verrucous skin lesion overlaying residual skin staples and an amputation stump of the second metatarsal bone on the dorsal foot. **Results:** The biopsy findings were inconclusive and suggested the necessity of distinguishing the lesion from verrucous carcinoma. The lesion was resected, and the residual skin staples were simultaneously removed. Investigation of the excisional biopsy confirmed our diagnosis of verrucous skin lesions on the feet in diabetic neuropathy. **Conclusions:** Verrucous skin lesions on the feet in diabetic neuropathy is often difficult to distinguish from verrucous carcinoma; in this case, the unusual location of the lesion could be attributed not only to sensory loss but also to the presence of an amputation stump and the persistence of the residual skin staples.

Verrucous skin lesions on the foot in diabetic neuropathy (VSLDN) was first described by Gerbig and Hunziker^1^ in 1995. Clinically, this condition is usually observed on protrusion sites such as the first metatarsal head, edge of skin grafts, plantar surface of the big toe, and calcaneal region in diabetic feet with neuropathy. The pathogenesis of VSLDN remains unknown; however, because it generally occurs in weight-bearing regions, chronic mechanical stimulation, such as friction or pressure, is suspected to play a key role.^1^^-^3 Accordingly, conservative treatments such as topical use of 5-fluorouracil, vitamin D_3_, or use of a hollowed-out sponge are generally recommended instead of surgical treatment.^4^^-^6 The pathological characteristics of VSLDN include pseudoepitheliomatous or pseudocarcinomatous hyperplasia, which can make it difficult to distinguish VSLDN from verrucous carcinoma (VC), an uncommon type of squamous cell carcinoma,^3^^,^^7^ which requires surgical treatment. Indeed, Ichimiya et al^2^ reported that some cases diagnosed and treated as VC may in fact be VSLDN.

We here report on a rare case involving a patient with a cauliflower-shaped hyperkeratotic nodule measuring 20 × 22 mm at an unusual non–weight-bearing site. The lesion was overlaying residual skin staples and an amputation stump of the second metatarsal bone; a biopsy specimen was needed to differentiate the lesion from VC, and it was successfully treated surgically.

## CASE REPORT

**Figure 1:**
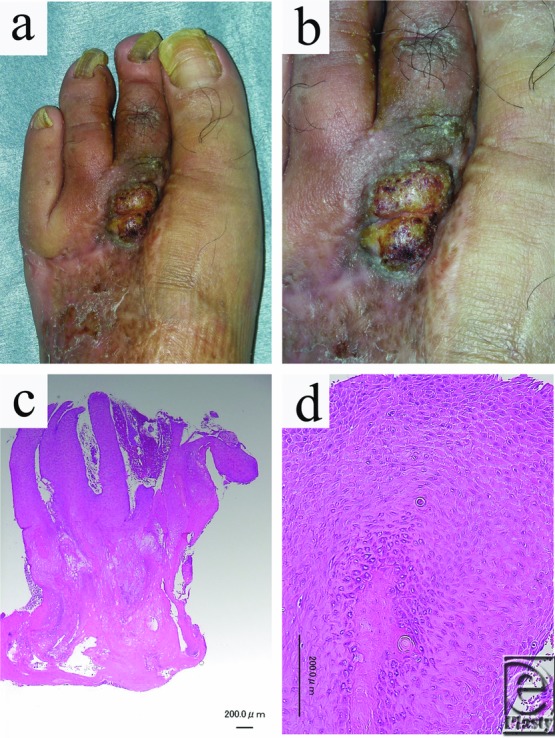
Clinical and histological findings. (a) A verrucous skin lesion on the dorsum of the left third metatarsal head. (b) Close-up of the lesion. (c) Skin biopsy specimen showing epidermal hyperplasia and elongation of the rete ridges. (d) Individual cell keratinization and mild atypism were observed.

A case report of a 58-year-old man with a 5-year history of diabetes mellitus who had been suffering from neuropathy for several years is presented. The second toe of his left foot had been amputated by an emergency physician due to gas gangrene. Seventeen months later, a verrucous lesion with partial ulceration was diagnosed on the dorsum of the third metatarsal head. Consequently, the patient was referred to our department, where a physical examination revealed a cauliflower-shaped hyperkeratotic nodule measuring 20 × 22 mm (Figs 1*a* and 1*b*). The surface of the lesion was partially ulcerated, with concomitant effusion from the ulcer. The lesion was located at the border between the normal and grafted skin, and the third metatarsophalangeal joint was contracted.

**Figure 2:**
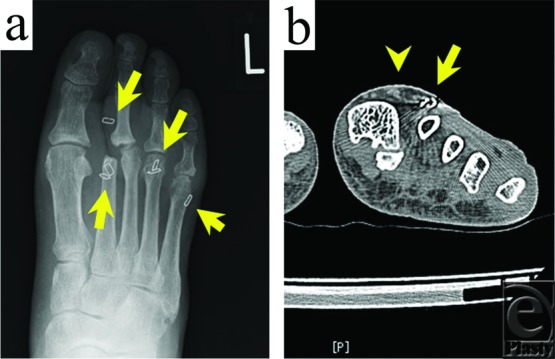
Radiograph and computed tomographic scan. (a) Radiograph showing the presence of skin staples (arrow) at the amputation stump. (b) Computed tomographic scan showing the lesion (arrowhead) overlaying a residual skin staple (arrow); no tumor invasion to the surrounding tissue was evident.

Squamous cell carcinoma was suspected, and a biopsy was consequently carried out. Histologically, epidermal hyperplasia and elongation of the rete ridges were observed, and mild atypism was recognized at the basal layer (Figs 1*c* and 1*d*), thus necessitating differentiation between VC and pseudocarcinomatous hyperplasia. Several skin staples used in the previous skin grafting were detected on the radiograph of the left foot (Fig 2*a*), and the third toe was dislocated toward the amputated site. Computed tomographic scans showed that the skin lesion was overlaying the residual skin staples; however, no tumor invasion to the surrounding tissues was observed (Fig 2*b*).

**Figure 3:**
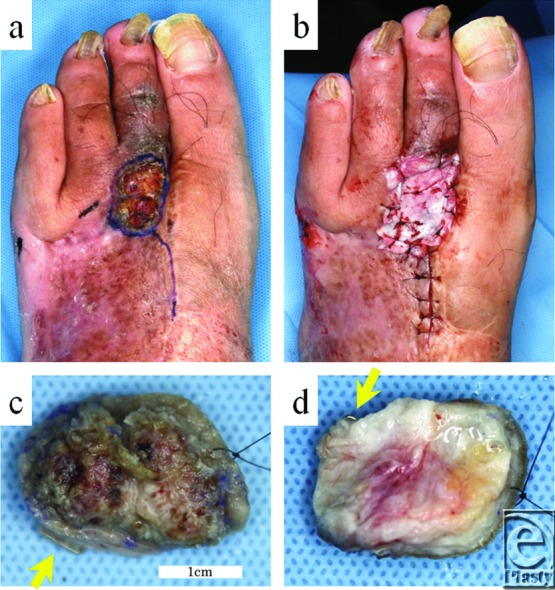
Preoperative and postoperative findings. (a) Resection with a 1-mm horizontal margin was performed. (b) Artificial dermis placed onto the skin defect. (c and d) Resected verrucous nodule with skin staples (arrow).

The lesion, along with a 1-mm margin (Fig 3*a*), was resected concurrently with the partial second metatarsal bone, and artificial dermis was placed onto the defect (Fig 3*b*). Moreover, the residual skin staples found just beneath the lesion (Figs 3*c* and 3*d*) were removed.

**Figure 4:**
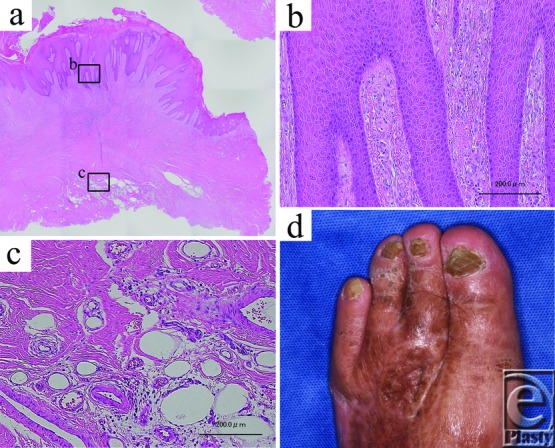
Histopathology of pseudocarcinomatous hyperplasia. (a) Epidermal hyperplasia and elongation of the rete ridges were observed. (b) No atypia was noted in the elongated rete ridges. (c) Invasion of inflammatory cells was recognized around the skin-stapled site. (d) Two years after the last operation.

Histologically, epidermal hyperplasia, elongation of the rete ridges, and invasion of inflammatory cells (Figs 4*a*–4*c*), features characteristic of pseudocarcinomatous hyperplasia, were observed. Thus, the lesion was diagnosed as VSLDN on the basis of histological findings, the anamnestic diabetic neuropathy, and the easily weight-bearing protruded location. As VSLDN is a benign disease and additional resection was not needed, skin from the groin region was grafted onto the skin defect. Figure 4*d* shows the status of the foot 6 years after the last operation. No recurrence has been observed to date.

## DISCUSSION

Verrucous skin lesions on the foot in diabetic neuropathy was first described in 1995 by Gerbig and Hunziker.^1^ Before this, Patki^8^ reported a case of a verrucous lesion in the ankles of a patient with Hansen's disease, in whom peripheral sensory function was decreased or lost. Gerbig and Hunziker^1^ reported that similar intractable verrucous lesions on weight-bearing regions, causing sensory disturbance, were seen in diabetic patients. Pathologically, VSLDN has been described as pseudoepitheliomatous or pseudocarcinomatous hyperplasia. While VSLDN is not a rare skin lesion, our literature review identified only 6 case reports in MEDLINE^1^^-^6; all these cases showed similar clinical and pathological findings and were believed to be caused by chronic stimulation in the region of sensory loss or reduction.

Biopsy is necessary to make the diagnosis of VSLDN; however, no standard treatment currently exists. Some studies have reported on the use of repeated shaving of the keratotic lesions, cryotherapy, basic fibroblast growth factor spray,^3^ combined topical use of 5-fluorouracil and tretinoin or vitamin D_3_,^4^ topical use of maxacalcitol,^5^ and wearing a hollowed-out sponge^6^; many of these studies do not recommend surgical management of these lesions. However, the reason for this recommendation has not been described.

The pathological findings of VSLDN highly resemble those of VC.^2^^,^^3^^,^^7^^,^^9^ Especially, for pathology specimens from small biopsy samples, pathologists sometimes face difficulty distinguishing VSLDN from VC. In these cases, surgical treatment is necessitated, and, indeed, foot amputation was performed in one such reported case.^3^ In our case, because we similarly had difficulty distinguishing VSLDN from VC based on the biopsy specimen, we decided to resect the lesion and carefully evaluate the whole specimen pathologically. Accordingly, the surgical margin was set to 1 mm and artificial dermis was placed on the defect. We have been following the patient for 6 years, and no recurrence has been observed to date. On the basis of this case, we believe that surgical treatment represents a valuable treatment option, especially for intractable cases and cases in which there is some difficulty distinguishing the lesion from VC.

The pathogenesis of VSLDN remains unknown. Some reports have discussed that because VSLDN occurs in feet with neuropathy, the formation of VSLDN may be associated with the amount of substance P^3^, whereas others have hypothesized a relation with vascular diseases.^10^ However, none of these hypotheses have yet been proven. Currently, chronic mechanical stimulation, such as friction or pressure, is regarded as the most convincing cause owing to the fact that VSLDN generally develops in weight-bearing regions.

In contrast, in our case, VSLDN occurred in a non–weight-bearing region, directly on top of residual skin staples. We hypothesize that the moderate infiltration of inflammatory cells around the skin-stapled site was associated with the formation of the slightly projected nodule and that this nodule promoted the formation of VSLDN through chronic mechanical stimulation, similar to callus formation. Indeed, the moderate infiltration of inflammatory cells around the skin-stapled site was recognized histologically (Fig 4*c*), and the inflammation exacerbated the fragility of the condition against chronic pressure and friction.

For gas gangrene, several incisions, as well as debridement, are urgently performed as lifesaving measures. After control of the infection, mesh skin grafting is often performed on the ulcer, as it is frequently extensive, and a stapler is used for the fixation of the graft. The use of skin staples is convenient and is associated with a reduced operative time. On the contrary, if the staples are left for a prolonged period, as in the present case, they can become buried inside the granulation and are difficult to remove. Thus, particularly on the feet, it is necessary to remove the staples carefully in order to avoid skin lesions such as VSLDN from developing, even in cases when the treated part is not a weight-bearing region.

## References

[1] Gerbig AW, Hunziker T (1995). Verrucous skin lesions on the feet in diabetic neuropathy. Br J Dermatol.

[2] Ichimiya M, Hamamoto Y, Muto M (2003). Verrucous skin lesions on the feet in neuropathy. J Dermatol.

[3] Sueki H, Furukawa N, Higo N, Akiyama M, Batchelor J, Iijima M (2004). Association of verrucous skin lesions and skin ulcers on the feet in patients with diabetic neuropathy. Clin Exp Dermatol.

[4] Sakai H, Fukami Y, Ibe M, Tamura T, Hashimoto Y, Iizuka H (1997). A verrucous lesion on skin grafted after necrotizing fasciitis in a diabetic patient successfully treated with combined topical 5-FU and tacalcitol. J Dermatol.

[5] Murao K, Oshima M, Miyajima O, Kubo Y (2012). Verrucous skin lesions on the feet in diabetic neuropathy successfully treated with topical maxacalcitol. Eur J Dermatol.

[6] Yamauchi K, Kobayashi T, Kawakubo C, Fujimoto N, Takahiro S, Tajima S (2014). Verrucous skin lesions on the feet in diabetic neuropathy: successful treatment using a hollowed-out sponge. Australas J Dermatol.

[7] Sarma D, Wang J, Bewtra C, Lee L (2006). Verrucous carcinoma arising in a chronic non-healing ulcer of the foot of a diabetic patient. Internet J Dermatol.

[8] Patki AH (1994). Verrucous skin lesions on the legs of leprosy patients. Br J Dermatol.

[9] Nakamura Y, Kashiwagi K, Nakamura A, Muto M (2015). Verrucous carcinoma of the foot diagnosed using p53 and Ki-67 immunostaining in a patient with diabetic neuropathy. Am J Dermatopathol.

[10] Dudek NL, Marks MB, Marshall SC (2006). Skin problems in an amputee clinic. Am J Phys Med Rehabil.

